# Inter- and intra-observer reliability of thoracic limb circumference measurement methods in sound dogs

**DOI:** 10.3389/fvets.2023.1172033

**Published:** 2023-08-14

**Authors:** Kelly Deabold, Jordan Harriz, Brandy Madeiros, Wendy Davies, Justin Shmalberg, Erin Miscioscia

**Affiliations:** Department of Comparative, Diagnostic and Population Medicine, University of Florida College of Veterinary Medicine, Gainesville, FL, United States

**Keywords:** outcome measures, forelimb, canine, rehabilitation, gulick, muscle mass

## Abstract

**Objective:**

The primary objectives of this study are to (1) compare the inter- and intra-observer reliability of thoracic limb circumference measurement methods in sound dogs, and (2) determine the most reliable thoracic limb positioning and location on the thoracic limb for performing circumferential measurements.

**Methods:**

Thoracic limbs of 10 apparently sound dogs (20 limbs) were blindly and independently measured by 3 observers. Triplicate measurements were performed with dogs in lateral recumbency at 50 and 70% brachial (Br) length (length between the greater tubercle and lateral humoral epicondyle) and 25% ABr length (length between the lateral humeral epicondyle and ulnar styloid process), both with the elbow extended and at an approximate weight-bearing (WB) angle. Intra-class correlation coefficients (ICC) with a 95% confidence interval (CI) were used for data analysis with a *p* < 0.05 being significant.

**Results:**

All measures had significantly good to excellent intra- (ICC 0.836–0.994, *p* < 0.001) and inter-observer reliability (ICC 0.834–0.996, *p* < 0.001). Inter-observer reliability was excellent at 25% ABr extended and WB positions, and at 50% Br WB position, with a wider confidence interval at the latter location. Intra-observer reliability was excellent across all observers for 25% ABr extended and WB, and 50% Br WB positions, also with a wider confidence interval at the latter location.

**Conclusion:**

Circumferential measurement of the canine thoracic limb was most reliable at 25% ABr length with the elbow either in an extended or WB position.

## Introduction

Objective outcome measures are an important part of veterinary physical rehabilitation to monitor patient progress and to evaluate treatment efficacy. Ideal measures have low variation and high reliability when compared between observers (1). Measuring limb circumference is a quick, easy, and inexpensive way to indirectly measure muscle mass, as opposed to other methods such as diagnostic ultrasound, computed tomography (CT), magnetic resonance imaging (MRI), or dual-energy x-ray absorptiometry (DEXA); however, limb circumference is also less specific and can be prone to high variation (2, 3). In humans, limb circumference is commonly used as an indirect method of evaluating muscle mass (4, 5). In dogs, thigh circumference has been shown to correlate with muscle mass of the pelvic limb (1, 6).

There are multiple variables that can affect limb circumference measurements, including the measuring device used and the observer performing measurements. In a study evaluating four commercial measuring devices, measurements taken with a tape measure and a retractable tape measure resulted in significantly smaller values than those taken with an ergonomic measuring tape and a circumference measuring tape (7). This study also evaluated intra- and inter-observer variation and recommended that sequential measurements be performed by the same individual and using the same device to reduce variability (7).

Additional variables affecting limb circumference measurements include the location of measurement on the limb, limb position, muscle engagement and tone. Canine thigh circumference measurements were found to be most reliable when performed at a distance of 70% of thigh length, when measured from the apex of the greater trochanter, with the stifle in an extended position and dogs in lateral recumbency (8). Canine thoracic limb circumference measurements obtained at 25% of antebrachial (ABr) length, had higher intra-tester reliability than measurements at 50% of brachial (Br) length, though elbow joint position was not reportedly standardized in this study (6).

Other variables to consider, especially when assessing dogs that have undergone surgery, include the effects of fur clipping and sedation or general anesthesia. Multiple canine studies have demonstrated that fur clipping leads to smaller thigh circumference measurements compared to the same unclipped limb (8, 9). Sedation did not result in significant variation in pelvic limb circumference measurements when compared to relaxed dogs in lateral recumbency (8, 10).

The development of a standardized measurement protocol for canine thoracic limb circumference measurement would optimize use of this outcome measure in clinical patients and research studies, similar to what has been determined for canine thigh circumference measurements. Few studies have investigated thoracic limb circumference and the reliability of measurement techniques (6–10). Locations of canine thoracic limb circumference that have been individually assessed so far include 50 and 70% the length of the Br, and 25% the length of the ABr, all measured from the proximal anatomic landmark (6, 10). Intra- and inter-observer reliability of circumferential measurements for these three locations along the canine thoracic limb have not yet been compared, nor has the effect of thoracic limb positioning, such as having the elbow extended versus in an approximate weight-bearing angle (WB) position.

The primary objectives of this prospective, blinded study are to (1) compare the inter- and intra-observer reliability of thoracic limb circumference measurement methods in sound dogs, and (2) determine the most reliable thoracic limb positioning and location on the thoracic limb for performing circumferential measurements.

It is hypothesized that canine thoracic limb circumferential measurements will have good to excellent reliability at the level of the proximal (25%) ABr with the elbow in an extended position.

## Materials and methods

### Study design

This study was designed as a prospective, blinded study using 3 observers of varied years of experience performing limb circumference measurements: Observer 1 (OB1, 5+ years of experience), Observer 2 (OB2, 2 years of experience), and Observer 3 (OB3, <1 year of experience). The study was reviewed and approved by the Institutional Animal Care and Use Committee (IACUC) of the University of Florida (IACUC# 202111538). Written informed consent was obtained from owners for the participation of their dogs in this study.

### Study dogs

Ten apparently sound, staff-owned dogs were recruited from the University of Florida’s Small Animal Hospital, and measurements were performed on both thoracic limbs (20 total limbs). All study data was obtained between February 24th and June 8th, 2022. To be included in the current study, dogs were required to be >1 year old, between 7–40 kg (15–88 lbs.), and have a short to medium length hair coat. Orthopedic and neurologic examinations were performed by either a diplomate or resident of the American College of Veterinary Sports Medicine and Rehabilitation to confirm that there were no orthopedic nor neurologic abnormalities in either thoracic limb and that dogs were apparently sound on visual gait analysis. Exclusion criteria included: historic thoracic limb lameness, abnormal orthopedic or neurologic examination of the thoracic limbs, chondrodystrophic breeds, and dogs that were poorly behaved or stressed in lateral recumbency with light manual restraint (i.e., excessive panting or wiggling, vocalizing, trying to bite). Dogs with historic pelvic limb abnormalities were included in the study if no lameness was appreciated on physical examination; however, the data from this subset of dogs was excluded for evaluation of a secondary study objective, comparing right to left limb circumferences, to avoid this confounding variable.

### Data collection

Dogs were gently, manually restrained in lateral recumbency, without chemical sedation, by one of two veterinary technicians certified in veterinary rehabilitation. Lateral recumbency was chosen based on results of previous studies of similar design and so that differences in muscle engagement during standing was eliminated (6, 8). The 3 observers were blinded from each other by performing measurements in a separate examination room without the other observers present, as well as from their own measurements by having one of the two technicians read and record the measurements. Observers alternated performing measurements in a randomized order based on observer availability.

**Figure 1:**
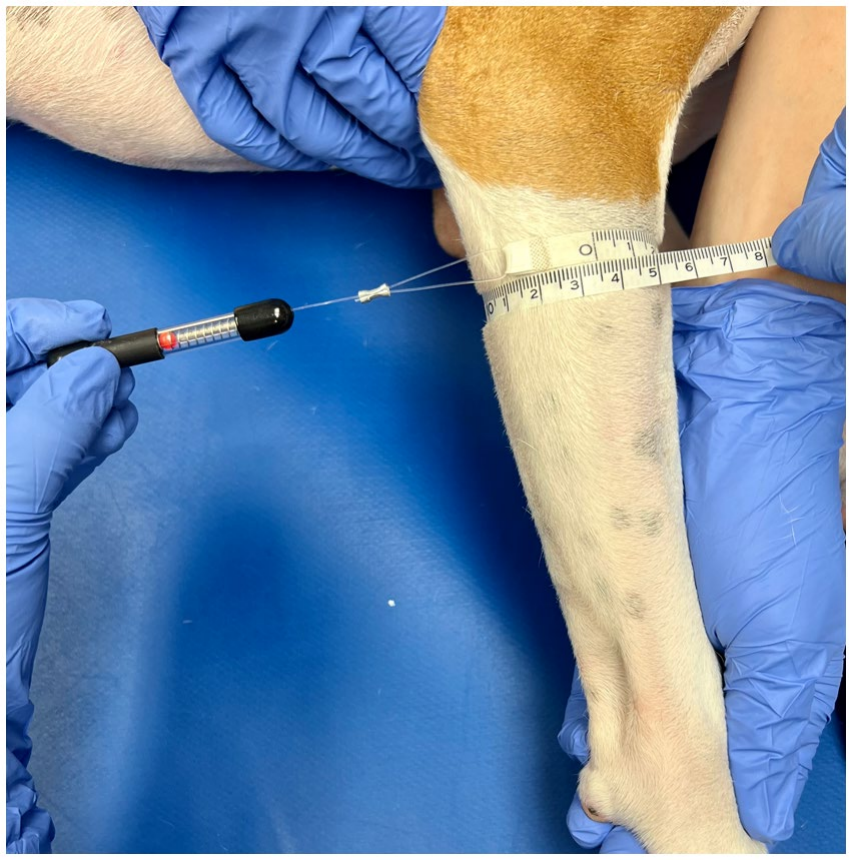
A gulick II tape measure was used to determine thoracic limb circumference for the brachium and antebrachium (shown here). The tape was placed around the limb with a consistent amount of end-tension and pulled taut until one of the red balls was completely exposed (2 oz. of end tension).

**Figure 2:**
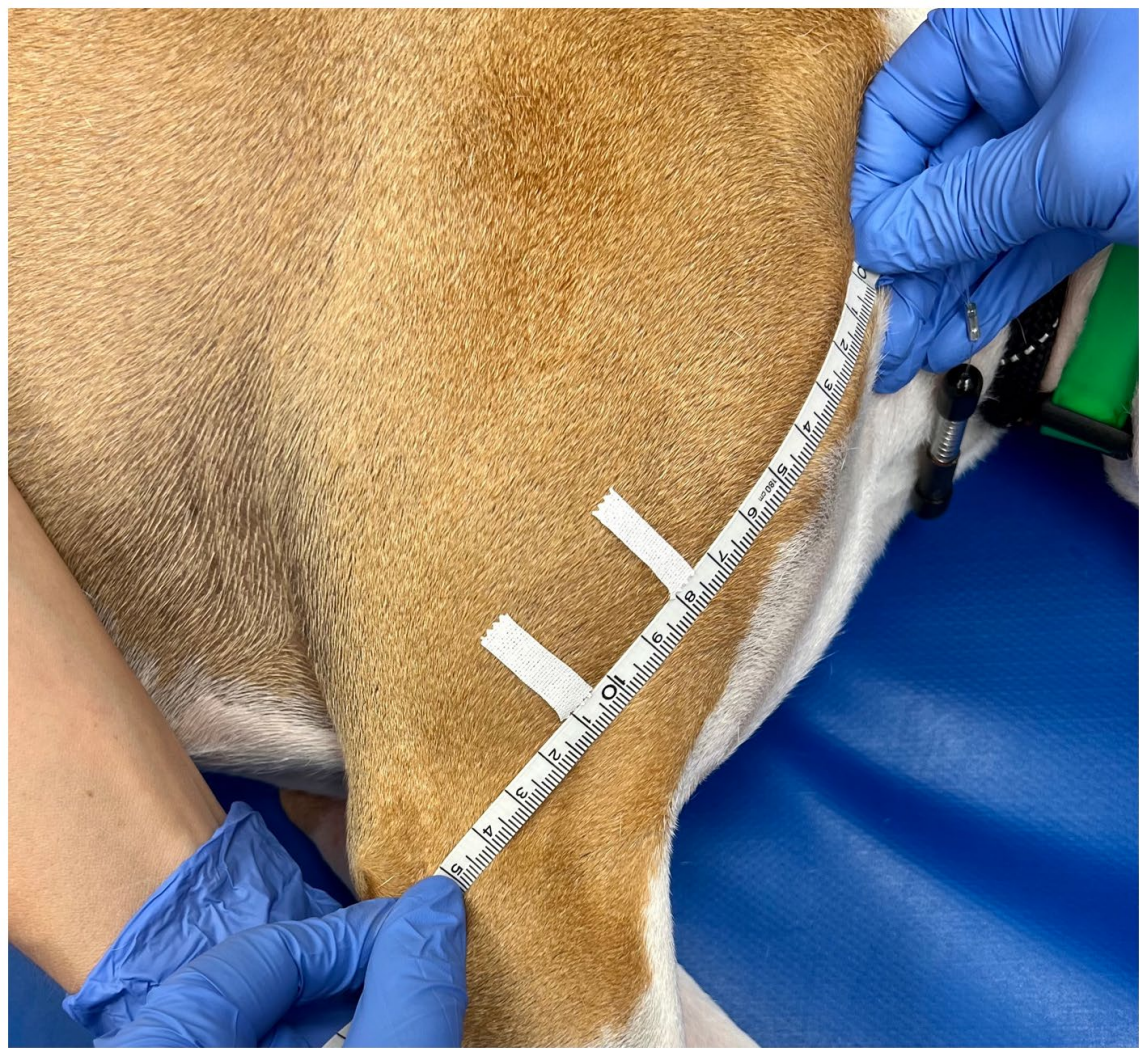
Brachial length was determined by measuring from the apex of the greater tubercle to the apex of the lateral epicondyle of the humerus. A small piece of half-inch white medical tape was used to mark points equal to 50 and 70% of the brachial length, as measured from the apex of the greater tubercle.

**Figure 3:**
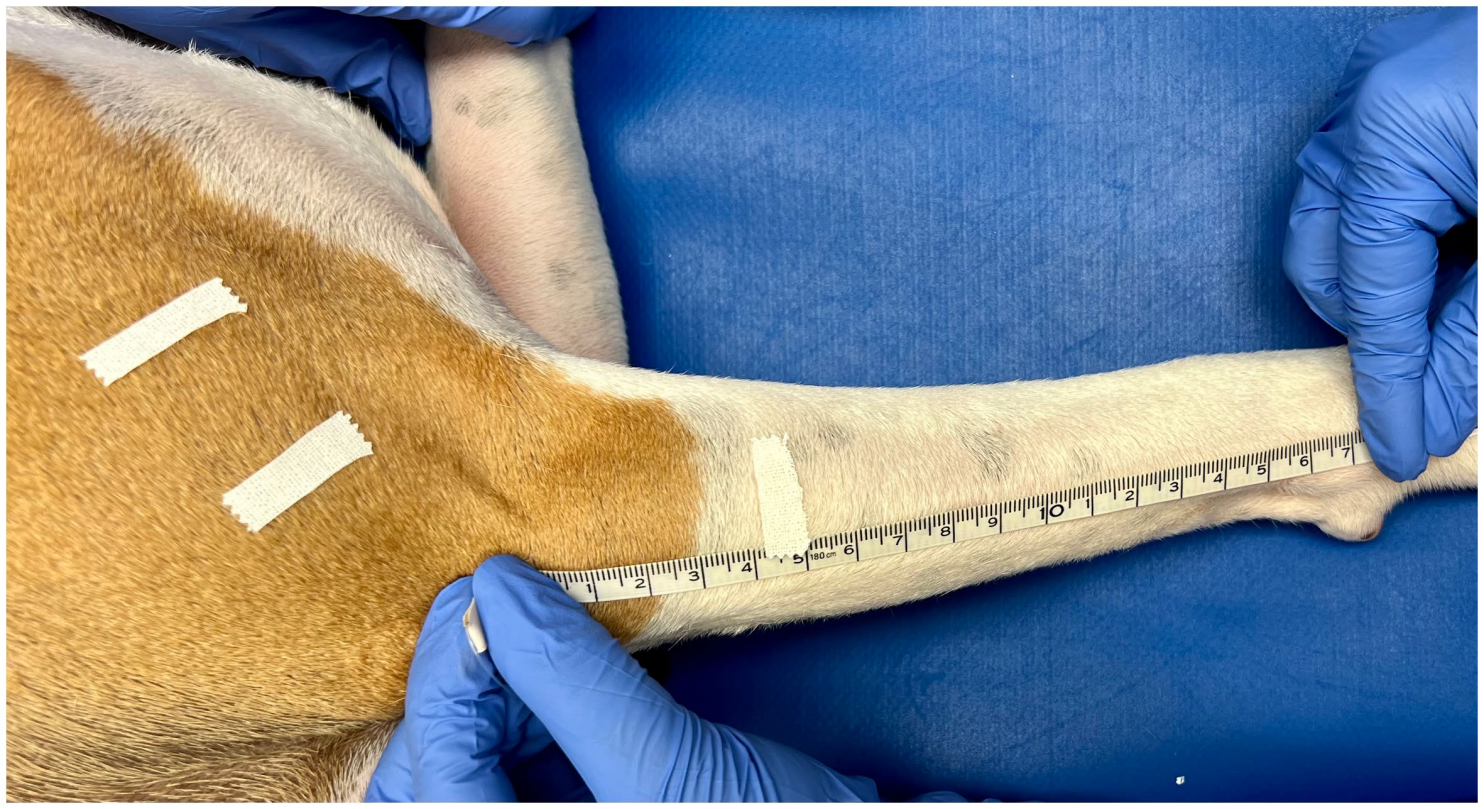
Antebrachial length was determined by measuring from the apex of the lateral epicondyle of the humerus to the apex of the ulnar styloid process. A small piece of half-inch white medical tape was used to mark points equal to 25% of the antebrachial length, as measured from the apex of the lateral epicondyle of the humerus.

Triplicate measurements were taken in centimeters using a gulick II tape measure (Sammons Preston, Gays Mills, WI, United States) by tightening the tape measure until the first of two indicator balls was exposed (2 oz. [56.7 g] of pressure; see Figure 1). The tape measure was removed from the limb and reset after each measure. Measurements were performed at 50 and 70% Br length (defined as the longitudinal length between the apex of the greater tubercle to the apex of the lateral epicondyle of the humerus) and 25% ABr length (defined as the longitudinal length between the apex of the lateral epicondyle of the humerus to the apex of the ulnar styloid process). A small piece of half-inch white medical tape was placed on the limb by the observer to mark the location of 50 and 70% Br length, as well as 25% ABr length (Figures 2, 3). The tape measure was positioned perpendicular to the long bone when obtaining the circumferential measures (Figure 1). At each of the lengths mentioned above, measurements were taken with the dog in lateral recumbency, and the limb held with the elbow in an extended, and then an approximated weight-bearing angle (WB) position.

Triplicate measurements were performed by all observers using all aforementioned measurement methods. Measurements were taken by each observer in the morning (AM), and again in the afternoon (PM), on the same day for one thoracic limb per dog. This was repeated on a different day for the contralateral limb to avoid prolonged restraint of study dogs on a single day. The side measured first was chosen at random. The number of days between measurements was varied based on owner availability. A total of 216 data points were collected per dog, or 108 data points per limb. The mean of the triplicate measures was calculated for each measurement method performed by each observer. Mean values were calculated following the completion of all measurements and utilized for statistical analysis. For inter-observer reliability, both the means of AM and PM measurements were used and reported in statistical analysis.

### Statistical analysis

Analyses of inter-observer and intra-observer measurement reliability were conducted using intra-class correlation coefficients (ICC) and corresponding 95% confidence intervals (95% CI). The interpretation of the quality of ICC was based on previously established categories for expressing levels of reliability as follows: high reliability, 0.90–0.99; good reliability, 0.80–0.89; fair reliability, 0.70–0.79; moderate reliability, 0.69–0.59; and poor reliability, <0.59 (6). For the most reliable measurement method(s) as determined by ICCs, a two-sided *t*-test was used to compare right versus left limb measurements. A *p* value of <0.05 was considered significant.

## Results

### Study dogs

Ten dogs met the inclusion criteria, including, 5 neutered males, 4 spayed females, and 1 intact female. Dogs were of various short to medium haired breeds, with most dogs being mixed breeds with short hair coats. Mean (±SD) age was 5.95 ± 3.47 years (median: 4.5 years, range: 2–12 years). Mean (±SD) weight was 23.96 ± 8.57 kg (median: 21 kg, range: 16.9–38.6 kg). Mean (±SD) number of days between first and second limb measurements was 12.1 ± 14.74 days (median: 7 days, range: 1–47 days). Four of the ten dogs had pelvic limb conditions including: 1 dog with a historic left femoral fracture, 1 dog with a historic right femoral head and neck ostectomy, 1 dog with historic left tibial plateau levelling osteotomy, and 1 dog with historic bilateral extracapsular repairs. These dogs were excluded from the secondary study objective of comparing right versus left thoracic limb circumferential measurements.

### Circumferential thoracic limb measurement reliability

All circumferential thoracic limb measurements had significantly good to excellent intra- and inter-observer reliability, as summarized in Tables 1, 2. Inter-observer reliability was excellent at 25% ABr extended and approximate WB positions and at 50% Br approximate WB position. The 50% Br approximate WB position had a wider 95% CI compared to both the 25% ABr extended and approximate WB positions. Inter-observer reliability was good at 50% Br extended position and 70% Br extended and approximate WB positions.

**Table 1:** Inter-observer reliability for canine thoracic limb circumferential measurement methods.

Location	Elbow position	Time	ICC (Avg measures)	95% CI
25% ABr	WB	AM	0.992	0.983–0.996
		PM	0.989	0.976–0.995
	Extended	AM	0.986	0.971–0.994
		PM	0.996	0.991–0.998
50% Br	WB	AM	0.904	0.537–0.970
		PM	0.906	0.618–0.969
	Extended	AM	0.876	0.614–0.955
		PM	0.854	0.506–0.948
70% Br	WB	AM	0.893	0.732–0.957
		PM	0.886	0.721–0.955
	Extended	AM	0.834	0.637–0.930
		PM	0.836	0.524–0.939

ICC, intra-class correlation coefficients; CI, confidence interval; ABr, antebrachial; Br, brachial; WB, approximate weight-bearing angle; AM, morning; PM, afternoon, value of *p* < 0.001.

**Table 2:** Intra-observer reliability for canine thoracic limb circumferential measurement methods.

Location	Elbow position	Observer	ICC (Avg measures)	95% CI
25% ABr	WB	OB1	0.991	0.978–0.997
		OB2	0.981	0.951–0.992
		OB3	0.991	0.978–0.996
	Extended	OB1	0.994	0.985–0.998
		OB2	0.965	0.913–0.986
		OB3	0.991	0.977–0.996
50% Br	WB	OB1	0.962	0.905–0.985
		OB2	0.941	0.851–0.977
		OB3	0.919	0.799–0.968
	Extended	OB1	0.926	0.816–0.971
		OB2	0.891	0.724–0.957
		OB3	0.917	0.792–0.967
70% Br	WB	OB1	0.935	0.837–0.974
		OB2	0.939	0.846–0.976
		OB3	0.871	0.676–0.949
	Extended	OB1	0.912	0.782–0.965
		OB2	0.842	0.596–0.938
		OB3	0.836	0.596–0.935

ICC, intra-class correlation coefficients; CI, confidence interval; ABr, antebrachial; Br, brachial; WB, approximate weight-bearing angle, value of *p* < 0.001.

Intra-observer reliability was excellent across all observers for 25% ABr extended and approximate WB, and 50% Br approximate WB positions. The 50% Br approximate WB position again had wider 95% CIs compared to both the 25% ABr extended and approximate WB positions. Intra-observer reliability was varied among observers (good or excellent) for all other measurements. OB1, the observer with the highest experience level, had excellent intra-observer reliability for all measurements, while OB2 and OB3 had good or excellent intra-observer reliability.

### Thoracic limb length measurement reliability

Both Br and ABr measurements had significantly good inter-observer reliability and excellent intra-observer reliability, as summarized in Tables 3, 4.

**Table 3:** Inter-observer reliability for canine thoracic limb length measurements.

Location	Time	ICC (single)	95% CI
ABr	AM	0.888	0.616–0.961
	PM	0.816	0.571–0.925
Br	AM	0.862	0.677–0.944
	PM	0.894	0.686–0.961

ICC, intra-class correlation coefficients; CI, confidence interval; ABr, antebrachial; Br, brachial; AM, morning; PM, afternoon, value of *p* < 0.001.

**Table 4:** Intra-observer reliability for canine thoracic limb length measurements.

Location	Observer	ICC (single)	95% CI
ABr	OB1	0.967	0.919–0.987
	OB2	0.917	0.804–0.966
	OB3	0.998	0.994–0.999
Br	OB1	0.986	0.965–0.994
	OB2	0.982	0.954–0.993
	OB3	0.979	0.947–0.992

ICC, intra-class correlation coefficients; CI, confidence interval; ABr, antebrachial; Br, brachial, value of *p* < 0.001.

### Right vs. left thoracic limb circumferential measurements

The measurement methods with excellent inter-observer reliabilities were used for comparison. The 4 dogs with historic pelvic limb conditions were excluded from this dataset analysis. There was no significant difference between right and left limbs at 25% ABr circumferential measurements with the elbow in extension, with the elbow in an approximate WB position, nor at 50% Br circumferential measurements with the elbow in an approximate WB position, as summarized in Table 5.

**Table 5:** Right vs. left thoracic limb circumferential measurements.

Location	Elbow position	Right mean (cm)	Left mean (cm)	% Diff	Value of *p*
25% ABr	WB	16.06	15.91	0.94	0.418
	Extended	16.07	15.82	1.57	0.129
50% Br	WB	26.07	26.74	2.54	0.362

ABr, antebrachial; Br, brachial; WB, approximate weight-bearing angle; cm, centimeters; % Diff, percent difference.

## Discussion

Circumferential measurements of the canine thoracic limb were most reliable within and between observers at 25% ABr length with the elbow in an extended or approximate WB position, yielding partial acceptance of our hypothesis. The only Br measurement with excellent inter- and intra-reliability was at 50% Br with the elbow in an approximate WB position, though values were less reliable than the ABr values as shown by a wider CI (Tables 1, 2). It was also noted by all observers that ABr measures were easier to obtain allowing for more reliable circumferential measurements. These findings support those of a previous study, which found that circumferential measures of the proximal ABr were reliable (6). Based on the findings of the current study, it is recommended to take circumferential measurements at the level of 25% Abr length with the elbow in an extended or approximate WB position. If measures of one or more specific muscle groups in the Br are needed for patient tracking, then it is recommended to take circumferential measurements at the level of 50% Br with the elbow in an approximate WB position.

In this study, elbow positioning did not appear to affect ABr measures, but for both 50 and 70% Br measures, approximate WB elbow positioning was more reliable. The increased reliability of measures taken at the ABr as compared to the Br, and effect of positioning on Br measurements, could be due to the conical shape of the Br muscles causing slipping of the measuring tape. Slipping was noted by all observers during Br measurement data collection, making measurements more tedious and time consuming as compared to ABr measurements. Having the elbow in an approximate WB position increases the muscle mass around the Br and may lead to less slipping of the measuring tape, and therefore more reliable measurements. The findings of the present study differ when compared to a prior study evaluating thigh circumference (8). In the prior study, the most reliable measure of thigh circumference was at 70% thigh length with the stifle in extension (8). This contrast could be due to conformational differences between the forelimbs and hindlimbs, differences in measurement protocols between the two studies, and/or different patient populations. Observers also noted that the Br circumferential measurements were impeded by the axillary fold during data collection. This may be another reason for decreased reliability in the Br region. This is similar to the findings of a previous study assessing thigh circumference in which observers found it to be more reliable to measure below the flank fold in the hindlimb (8).

In the present study, OB1 had the highest level of experience performing canine limb circumference measurements (5+ years) and the most reliable circumferential measures as compared to OB2 (2 years) and OB3 (<1 year), when considering all measurements. However, for 25% ABr (approximate WB and extended) and 50% Br (approximate WB) measurements, all observers had similarly excellent reliability. Based on these results, observers with 5 or more years of experience may perform circumferential measurements more reliably than observers with ≤2 years of experience. Another study assessing limb circumference in dogs did not show consistent differences between observers of different experience levels (7). In human medical literature, when assessing lower extremity girth, one study showed no difference between three observers with 7-, 5-, and 2 years’ experience (4). A more recent study from the human literature compared consistency and accuracy of measurement of lower limb amputees (11). In this study, students with less than 1 year experience were more consistent when taking linear measurements as compared to experienced practitioners but had much larger standard deviations in linear and circumferential measures. The experienced practitioners with a range of 2–28 years of experience were more consistent with circumferential measures with smaller standard deviations (11).

Inter-observer reliability was good for Br and ABr length measures and intra-observer reliability was excellent for Br and ABr length measures. Limb length can be variable based on the landmarks used and differences in palpation techniques. For this study, we defined Br length as the longitudinal location between the apex of the greater tubercle to the apex of the lateral epicondyle of the humerus and ABr length as the longitudinal location between the apex of the lateral epicondyle of the humerus to the apex of the ulnar styloid process. These landmarks have also been used in previous studies to assess forelimb circumference (6, 10). The results of this study show that each observer was consistent with their measurements and had good agreement with each other. The authors chose to have each observer measure limb length to most closely mimic what happens in clinical practice. If limb length had instead been measured and consistent across observers, it is possible this may have contributed to increased inter-observer limb circumference reliability. To the authors’ knowledge, this is the first study assessing the inter-observer and intra-observer reliability of Br and ABr lengths. Further studies with larger heterogeneous populations will be needed to correlate these findings.

When comparing right to left circumferential forelimb measurements, based on our data it could be expected that apparently sound dogs are symmetric with no statistically significant circumferential differences between limbs. These findings are in contrast to a previous study assessing mid-thigh circumference in dogs (7). In the Baker et al. study, the left thigh (mean: 31.4 cm ± 5.4) was larger than the right (mean: 30.3 cm ± 4.8) across all observers and measuring devices. It was also noted that the left was larger when assessing circumference of the tibial tuberosity, hock, and carpus (7). When evaluating symmetry of sound dogs using objective gait analysis and kinematics, it has been demonstrated that sound dogs can have asymmetry when comparing left and right limbs (12–15). When assessing healthy trotting dogs using force plate analysis, dogs had a percent difference in vertical symmetry indices of <8% for both thoracic and pelvic limbs (15). Further research is indicated to determine if there is a true expected variance in contralateral limb circumference measurements of sound dogs. This could impact clinician use of the contralateral ‘sound’ limb in lame dogs as a comparison tool to guide response to therapy.

Limitations of this study included the small, heterogeneous population of dogs evaluated, with variation in size, coat length, and body and muscle condition. There were multiple limitations due to the study design. While performing measurements, only the elbow angle was controlled and the WB angle was estimated rather than using goniometry to determine the actual WB angle. Differences in shoulder angles could have contributed to higher variation seen in the brachial circumferential measures as multiple muscle groups cross both the shoulder and elbow, though the authors attempted to approximate a neutral, WB position of the entire forelimb. Approximation of WB angle was elected to more closely align with what is likely to occur in clinical practice, though was hypothesized to result in reduced reliability when compared to measurements with the elbow in extension, which was ultimately rejected. Observers read limb length measurements themselves, thus limb length measurements were not blinded. A small piece of medical tape was used on the skin to mark where the Br and ABr circumferential measurements should be taken, however, the tape could have moved during data collection leading to variations in length and circumference.

In conclusion, circumferential measurement of the canine thoracic limb was the most reliable and easiest to perform at 25% ABr length with the elbow either in an approximate WB or extended position. Further research is needed in dogs of different conformations, long coat lengths, and with common disease conditions to ensure these findings remain applicable.

## Data availability statement

The raw data supporting the conclusions of this article will be made available by the authors, without undue reservation.

## Ethics statement

The animal studies were approved by The Institutional Animal Care and Use Committee of the University of Florida. The studies were conducted in accordance with the local legislation and institutional requirements. Written informed consent was obtained from the owners for the participation of their animals in this study.

## Author contributions

KD and EM participated in study design, data collection and analysis, and manuscript preparation. JH, BM, and WD participated in data collection. JS participated in data analysis and manuscript preparation. All authors contributed to the article and approved the submitted version.

## Conflict of interest

The authors declare that the research was conducted in the absence of any commercial or financial relationships that could be construed as a potential conflict of interest.

## Publisher’s note

All claims expressed in this article are solely those of the authors and do not necessarily represent those of their affiliated organizations, or those of the publisher, the editors and the reviewers. Any product that may be evaluated in this article, or claim that may be made by its manufacturer, is not guaranteed or endorsed by the publisher.

## Abbreviations

ABr: Antebrachium
Br: Brachium
WB: Weight-bearing
IACUC: Institutional Animal Care and Use Committee
ICC: Intra-class correlation coefficients
CI: Confidence interval
CT: Computed tomography
MRI: Magnetic resonance imaging
DEXA: Dual-energy x-ray absorptiometry
